# Gastric cancer in Central America: a scoping review

**DOI:** 10.3332/ecancer.2025.1834

**Published:** 2025-01-23

**Authors:** Jade Tso, Mustafa Al-Qaraghli, Susana Galeas, Mustafa Faleh Abidalhassan, Cameron E Gaskill

**Affiliations:** 1 Davis School of Medicine, University of California, Sacramento, CA 95817, USA; 2 Davis Department of Surgery, Division of Surgical Oncology, University of California, Sacramento, CA 95817, USA; 3 Clínica Esperanza, Roatán 34101, Honduras; 4 Northwell Health Department of Surgery, Lake Success, New York, NY 11042, USA

**Keywords:** gastric cancer, Central America, review

## Abstract

Stomach cancer is the second most common cause of cancer-related death in Central Latin America and the fifth most common cancer by incident in the region. Understanding the epidemiology of stomach cancer is crucial to the appropriate planning, implementation and evaluation of comprehensive cancer control programs. The objective of this scoping review was to quantify the population-based incidence of stomach cancer in Central America from available data, identify reported risk factors, presentation and oncologic stage and explore the frequency of treatment used and survival outcomes for stomach cancer in Central America. Primary reports, cancer registries, hospital registries, endoscopy registries, case studies and case series focusing on the epidemiology of gastric cancer in Belize, Costa Rica, El Salvador, Guatemala, Honduras, Nicaragua and Panama, along with its treatment modalities and mortality rates were included. After identifying 616 citations, 20 studies met the inclusion criteria and were selected for data extraction. 12 were from Costa Rica and 5 from Honduras, with few studies from other countries such as Belize, El Salvador and Panama. Crude rates of gastric adenocarcinoma varied widely across different studies, with rates ranging from 0.09/100,000 to 32.04/100,000 in Costa Rica between 1996 and 2015. Overall, there was a general decrease in crude rates over recent study periods. Studies in El Salvador and Panama reported lower crude rates compared to Costa Rica. Non-cardia cancers were more common than cardia cancers. Surgery was the main treatment discussed in the reviewed papers. Mortality data were limited. Our review highlights the need for reliable cancer registries in this region. Often, cancer registries provide the only opportunity for properly assessing the extent and nature of cancer burdens in developing countries. This information is crucial in creating priorities for cancer control public health programs.

## Introduction

Gastric adenocarcinoma (GC) is the third most common cause of cancer-related mortality worldwide and the leading infection-associated cancer [1, 2]. In 2019, there were an estimated 957,000 deaths and 1.27 million incident cases of stomach cancer worldwide. The burden of disease shows wide geographical variation, with more than 70% of total cases occurring in low and middle-income countries (LMICs) [3]. As a result of the various interacting risk factors for GC, the burden of disease is clustered into epidemiological pockets throughout the world, with emerging evidence that the largest pocket in the Western hemisphere is located in Central America. It is estimated that stomach cancer is the second most common cause of cancer-related death in Central Latin America and the cancer with the fifth most absolute number of incident cases in the region [1]. While there have been steady declines in GC worldwide in the past few decades [4] stomach cancer is evident to be a growing concern in Central America. The Central America Four (CA-4) region, including El Salvador, Guatemala, Honduras and Nicaragua is the largest low- and middle-income country region in the Western Hemisphere with a growing cancer burden that is expected to increase by 73% by 2030 [5]. Previous studies have observed that two-thirds of GC incident cases in the region are male and half of the patients come from rural areas [6]. The population of GC patients in Central America tends to be younger and the non-cardia and intestinal subtypes tend to be more common, which is similar to other low and middle-income regions [6]. The high prevalence of intestinal subtypes can be explained by earlier lifelong *H. pylori* infection as well as other host and environmental factors [6].

Understanding the epidemiology of stomach cancer is a crucial element of appropriate planning, implementation and evaluation of comprehensive cancer control programs. However, there are large disparities in the availability and quality of data on cancer burden globally. There is a paucity of literature regarding gastric cancer epidemiology in Central America with the exception of Costa Rican and Panamanian national cancer registries. Population-based cancer registries (PBCRs) and hospital-based cancer registries are especially limited in the CA-4.

The scoping review had multiple objectives. The first was to quantify the population-based incidence of stomach cancer in Central America from all available data. Second was to identify reported risk factors, presentation and oncologic stage for stomach cancer in Central America. Third was to explore the frequency of treatment used and survival outcomes for stomach cancer in Central America. This paper aimed to synthesise current literature describing the epidemiology of gastric cancer in Central America. We present this article in accordance with the preferred reported items for systematic reviews and meta-analyses (PRISMA)-ScR reporting checklist.

## Methods

We conducted a scoping review following Arksey and O’Malley’s [7] expanded framework to systematically search and explore research on gastric cancer incidence in Central America as well as treatment and outcomes. Research ethics approval was not required. This scoping review is reported according to the PRISMA and an extension of reporting scoping reviews, PRISMA-Scr [8]. This study was originally designed as a systematic review and registered through PROSPERO (PROSPERO 2022 CRD42022342989) [9].

### Research questions

1. What is the population-based incidence and age-standardised rate of stomach cancer in Central America?
2. What are the reported risk factors, associated symptoms and stage of presentation for stomach cancer in Central America?
3. What is the frequency of treatment used for stomach cancer in Central America?
4. What are the mortality rates for stomach cancer in Central America?

### Search strategy

We searched for relevant documents in PubMed, Embase, Web of Science, Global Health (CAB Direct) and the Latin American and Caribbean Health Sciences Literature databases. In consultation with a health sciences librarian, we developed a search strategy using MeSH headings for gastric cancer and Central America. The final search strategy for all databases can be found in Appendix 1.

### Study selection process

The inclusion and exclusion criteria for studies followed the population, concept and context categories for scoping reviews. Literature was included if they were primary reports, cancer registries, hospital registries, endoscopy registries, case studies and case series that described the epidemiology of gastric cancer in Central America, its treatment and outcomes (including survival and post-operative complication rates). Research and reports set in Belize, Costa Rica, El Salvador, Guatemala, Honduras, Nicaragua and Panama only were included. Nonprimary reports, consolidated data, systematic reviews and conference abstracts were excluded. Reports published over 25 years ago (before 1997) were also excluded. Reports available in English or Spanish were included.

An initial screen of titles and abstracts was conducted to determine eligibility. Duplicates were removed. All results were tracked using a systematic review platform, Covidence (Melbourne Australia). A reason for excluding each article after full-text review was recorded based on the eligibility criteria.

### Charting the data

After initial title and abstract screening, two authors (JT & MA) reviewed the full-text articles to determine inclusion/exclusion. Differences were resolved by discussion. The full texts of included studies were reviewed and data were extracted from eligible studies using a prestructured data collection form, with one team member extracting data and another team member checking the extracted data for reliability and accuracy. The following data were extracted: bibliographic information, country of study, location of study, study type, type of registry, total patients, number of gastric cancer cases, age-standardised ratio or crude rate (if given), listed risk factors, location of cancer, treatment provided, listed barriers to care and outcomes of treatment (survival rate and postoperative complication rate). To calculate crude rates for studies that only gave a number of cases of gastric cancer during a specified time period in a specified location, population data (including percentage of population by sex by year and total population by year) was pulled from publicly available databases or local censuses [10–12]

### Collating, summarising and reporting findings

Study characteristics were summarised using frequencies and percentages. Study findings were synthesised narratively. Qualitative and quantitative study findings were reported separately.

### Critical appraisal

Critical appraisals of each of the included papers were done according to criteria from the Joanna Briggs Institute [13].

## Results

**Figure 1.**
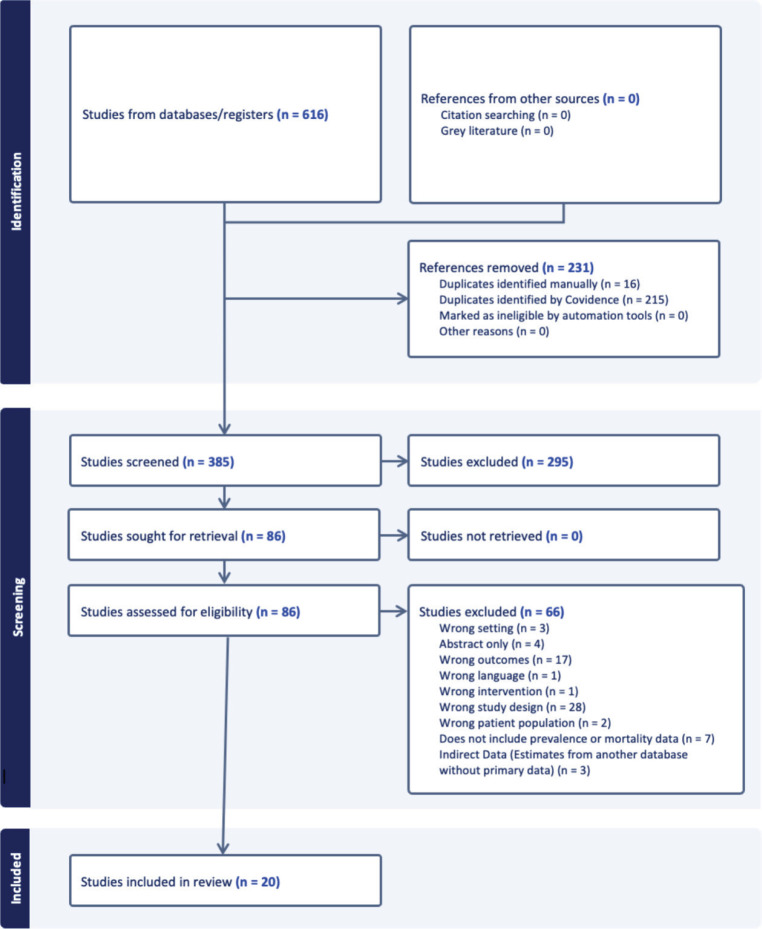
Study selection process.

The literature search yielded a total of 616 citations (Figure 1). After deduplication, 385 articles were included for screening. Following a review of titles and abstracts, a total of 83 articles that met initial inclusion criteria were included for a full-text review. Reference lists from these articles were examined for potential inclusion of additional citations. After a full-text review, 20 studies were included for data extraction.

### Study characteristics

Study characteristics are summarised in Table 1. Details on individual studies are provided in Table 2. The majority of studies were published between 2015 and 2020 (53%), *n* = 19. Six studies were cross sectional, four cohorts, three case control, one prevalence study, one cancer registry and three other study types. Other study types included a summary analysis of cancer registries, hospital registries, prospective population-based studies that used endoscopy and pathology registries, a non-randomised community control trial and a description of a screening program. 58% of the studies were focused in Costa Rica and 26% in Honduras. There was one paper that included data from Costa Rica, El Salvador, Honduras and Panama. Notably, none of the included studies described the epidemiology of gastric cancer in Belize. All included studies were assessed using the Joanna Briggs critical appraisal checklists and were deemed appropriate to include in this review.

### Crude rates

Crude rates were directly provided by authors in 1 paper [28]. The remaining papers included in Table 3 provided total GC cases for a certain time period. Total GC cases were divided by the sum of the population in the study’s catchment area over the time period reported. Rates were reported by 100,000 people and delineated by sex if allowed by data given in the original study.

As shown in Table 3, crude rates varied considerably by sex, country and time period studied. Overall, crude rates were higher in males than in females for all the studies in which rates could be separated by sex. Total crude rates in Costa Rica ranged from 0.09/100,000 to 32.04/100,000. Overall, crude rates appeared to decrease over more recent study periods. Total crude rates in Honduras ranged from 11.88/100,000 to 20.10/100,000, with the latter being from a more recent paper with a study period of 2013 to 2017. While this trend could be indicative of a potential true rise in rates in Western Honduras, it could also be indicative of registries becoming more robust and increased access to hospital services and the ability to be diagnosed. The single study conducted in El Salvador and a single study conducted in Panama yielded total crude rates of 2.09/100,000 and 3.96/100,000, respectively. Corral *et al* [6] provided case numbers for Guatemala, El Salvador, Honduras, Nicaragua, Costa Rica and Panama for the time period 1985 through 2011, with a calculated crude rate for the region of 2.65/100,000 for males and 1.56/100,000 for females.

### Characteristics of gastric cancer and listed risk factors

Twelve papers described the characteristics of gastric cancer in their study population. Six studies reported on anatomical sites–each study listed non-cardia as being the most common site with rates ranging between 35% and 100% of cases. Three studies reported Borrmann class, with type 3 being the most commonly reported with rates ranging from 48.88% to 66.19%. Histology varied among studies. Five studies reported intestinal pathological subtype to be more common and one reported diffuse to be more common, while two reported similar percentages of intestinal and diffuse pathologies. Five studies reported on risk factors and symptoms. Factors including male sex, older age, lower socioeconomic status and living in a rural area were reported as increasing risk for having GC. Antecedent symptoms such as vomiting, loss of appetite and stomach pain were also listed as risk factors. Lack of social security, a poorly differentiated tumour, clinical stage IV and overlapping anatomic location with an increased risk of dying from GC. Insurance coverage, geographic location and cultural differences were also listed as risk factors for mortality (Table 4).

### Treatments and listed barriers to care

Eight papers included information on GC treatment, with all including surgery as a provided treatment amongst their study population. Chemotherapy, radiation and palliative support were also described in five studies. Reported barriers to care included lack of social security, living in a rural or remote area, political turmoil and poverty (Table 5).

### Mortality rates

Six papers reported mortality rates, with five being from Costa Rica. Arevalo de Acevedo and Arenivar Franco [14] in El Salvador reported a mortality rate of 5/100,000 for males, 2.5/100,000 for females and 3.7/100,000 overall. The remaining four papers were based in Costa Rica. According to Mora Alvarado 2003 and 2006, mortality rates have overall decreased in the final two decades of the twentieth century (Table 6).

## Discussion

Our review identified a total of 20 publications discussing the epidemiology, treatment and outcomes of gastric cancer in Central America during the time period between 1997 and 2022. This points to the paucity of literature on this topic, particularly in Central American countries outside of Costa Rica. 12 of the 20 publications were from Costa Rica and 5 were from Honduras (with the majority coming from Western Honduras). Notably, there were no papers focusing on the epidemiology of GC in Belize alone and only one in El Salvador and one in Panama. Estimated crude rates were far ranging, particularly in Costa Rica for which there were the most studies available. The discrepancies in these rates may be attributable to limited data availability as well as studies representing a large geographical area – with some being nationwide versus others focusing on specific provinces. Total crude rates ranged from 0.09/100,000 to 32.04/100,000 in the time periods covered by the studies (1996–2015). Overall, crude rates appeared to decrease over more recent study periods. This is consistent with global trends of GC declining over the past three decades [33].

Overwhelmingly, non-cardia cancers were found to be more common than cardia cancers in the region. This is expected given the strong positive association between *H. pylori* and non-cardiac gastric cancer. A previous study found the odds ratio for the association between *H. pylori* and non-cardiac gastric cancer to be 4.79, consistent across sociodemographic, clinical and lifestyle factors [34] A recent study done in Western Honduras and Central Guatemala found *H. pylori* seropositivity to be 87% with 83% of the nearly 1,150 subjects to have active *H. pylori* infection [35]. This finding also may explain the observed decreased incidence of GC in the region, as improved access to clean water and sanitation decreases *H. pylori* infections.

The incidence and mortality rates of this study were similar to those found in previous studies. Sierra *et al* [36] compiled crude rates and mortality rates in Central and South America using population-based registries and nation-wide cancer deaths from the World Health Organisation’s mortality database up to 2007. This study found that crude rates in Costa Rica were 22.0/100,000 and 14.4/100,000 for males and females, respectively, during the 2003–2007 period, consistent with our review [36]. El Salvador had crude rates of 2.8/100,000 for males and 1.8/100,000 for females in the 1999–2003 period, close to our total crude rate of 2.09/100,000 total crude rate [36]. Mortality rates in El Salvador differed: our review found 3.7/100,000 for males and 2.5/100,000 for females, while Sierra *et al* [36] reported 8.8/100,000 for males and 7.6/100,000 for females. In Costa Rica, Sierra *et al* [36] reported crude death rates at 17.1/100,000 for males and 9.4 for females from 2003 through 2007, while studies in our review found rates that varied from 8/100,000 to 62.6/100,000, likely due to the broader time period studied. Similar to Sierra *et al* [36], our review also found non-cardia cancers to be more common than cardia cancers.

Our findings were similar to previous studies that pulled from World Health Organisation cancer registries. Variations amongst crude rates and crude death rates between studies can likely be attributed to the incompleteness of the data available. The overall geographic and temporal variation of stomach cancer rates observed in this review may be explained by variations in the prevalence of *H. pylori*, improvements in sanitation, preservation and storage of foods and smoking patterns [33] Additionally, the included studies may have underestimated incidence of gastric cancer–in absence of a systematic screening program for GC, only symptomatic cases are detected and asymptomatic cases may go unreported. For patients with limited access to healthcare, particularly those living in poverty or those living in rural areas, symptomatic patients may never make it to a health facility and their cases may go unreported [20].

The crude rates and mortality rates found in the current study point to the emerging epidemiological hotspot for GC in Central America. Comparatively, East Asia has the highest incidence rates of GC for both males and females at 32.5 and 13.2 per 100,000, respectively. While the rates seen in Central America are not as high as East Asia, they remain significantly higher than in North America (5.4 per 100,000 for males and 3.1 per 100,000 for females) [37]. Mortality rates for GC are also estimated to be higher in Central America than North America [37].

Our review highlights the need for reliable cancer registries in the Central American region. Often, cancer registries provide the only opportunity for properly assessing the extent and nature of cancer burdens in developing countries. Population-based GC registries are valuable for identifying demographic and geographic differences in incidences and mortality rates [38]. Hospital-, clinic- or laboratory-level data give more details for public health officials to understand local epidemiology and risk factors [38]. However, there are a number of hurdles that exist to advancing the collection of this data in LMICs including traditional Ministry of Health priorities, budget constraints and pathology service limitations [6]. In the Central Latin American context, cancer registry success may be possible by focusing attention on local-level data collection, with the Western Honduras Copán registry serving as a good example. These local registries can be monitored by national data collection guidelines [38]. Investing in GC surveillance programs and training health workers to collect accurate data can prove to be a valuable investment for regional and national health systems. This information is crucial in creating priorities for cancer control public health programs [39].

Eight of the 20 included studies discussed treatment, and surgery was provided to patients. Studies have described substantial barriers to care for GC in Central America, particularly in rural areas of the CA-4. Estevez-Ordonez *et al* [22] found that only 1 in 5 patients received curative or palliative surgical treatment, and 1 in 12 patients received chemotherapy in rural western Honduras, with living in households below the regional poverty standard and distance from a facility offering chemotherapy listed as major barriers [22]. Mortality data were scarce, only included in 5 of the 20 studies, with crude death rates being far-ranging. These factors point to the need for increased capacity in the region, both in terms of being able to provide far-reaching surgical treatment as well as chemotherapy, particularly to rural patients.

This paper is the first to compile published data regarding the epidemiology and treatment of GC in Central America using primary reports. While one of the strengths of this paper is that it highlights the need for additional research in regard to this topic, the small number of publications (20) limits the ability to do subsequent meta-analysis of GC incidence, treatment or outcomes in the region. Additionally, as the majority of the papers did not give a calculated crude rate, the authors of the current manuscript used the number of GC cases stated in the papers to calculate crude rates from publicly available population data. This additional step of estimating crude rates introduces a margin of error that accompanies using population data estimates.

## Conclusion

Stomach cancer is one of the most frequently diagnosed cancers and the leading cause of cancer death in Central America, with current literature pointing to the region becoming an emerging hotspot. The current literature review is the first of its kind to consolidate information on epidemiology, treatment and mortality rates of GC in Central America from existing literature over the past 25 years. The crude rates we calculated and mortality rates found from our review were comparable to the rates calculated in other studies using WHO registries. The authors of the studies we pulled from also discussed similar known risk factors including age, sex, low socioeconomic factors and living in a rural area. This review highlights the need for increased data collection on gastric cancer prevalence, treatment and outcomes in Central America.

## Conflicts of interest

The authors declare no conflicts of interest.

## Funding

The authors declare no funding sources for this study.

## Figures and Tables

**Table 1.** Characteristics of included studies.

Date	
1997–2002	1
2003–2008	5
2009–2014	2
2015–2020	11
2021–2022	1
Study design	
Cross sectional	8
Cohort	4
Case control	3
Prevalence study	1
Cancer registry	1
Other	3
Country	
Belize	0
Costa Rica	11
El Salvador	1
Honduras	5
Panama	1
Multiple countries	1

**Table 2.** Individual study information for included articles.

Lead author	Year	Country	Location	Study design	Aim of study	Population description	Number of gastric cancer cases
Arevalo de Acevedo and Arenivar Franco [14]	2019	El Salvador	Instituto Salvadoreno del Seguro Social	Cross sectional study	To determine the epidemiological profile and hospital mortality due to gastric cancer in Institute for Salvadoran Social Security patients between 2000 and 2017.	Patients enrolled with the Institute for Salvadoran Social Security with gastric cancer (defined by ICD-10 C16 and C169.)	2,295
Barrantes Solís [15]	2009	Costa Rica	San Vicente de Paúl Hospital in Heredia Province, Costa Rica	Cross sectional study	To analyze the main causes of cancer in patients at the San Vicente de Paúl Hospital and determine their frequency according to the patient's place of origin	Cancer patients discharged from San Vicente de Paúl Hospital	171
Castro et al [16]	2017	Panama	National Oncology Institute of Panama	Cohort study	To evaluate the association between socioeconomic and clinical variables with survival, describe the survival outcomes according to clinical stage and estimate the direct costs associated to GC care in a Panamanian population with GC.	Patients treated at the National Oncology Institute, the leading public institution for cancer treatment in Panama, who were diagnosed with GC (ICD-10 C16.0–C16.9)	611
Cordero-García *et al* [17]	2019	Costa Rica	Four major research hospitals in Costa Rica (Hospital San Juan de Dios, Hospital Rafael Ángel Calderón Guardia, Hospital Max Peralta and Hospital México)	Cohort study	To evaluate the impact of pharmacological therapies in the survival of patients diagnosed with metastatic gastric cancer from Costa Rica.	Patients with metastatic gastric cancer who were treated with pharmacologic therapy at Hospital San Juan de Dios, Hospital Rafael Ángel Calderón Guardia, Hospital Max Peralta and Hospital México. (These three hospitals were all the hospitals in Costa Rica with Clinical Oncology Service and Oncology Pharmacy at the time of the study.)	168
Cordero-García *et al* [18]	2018	Costa Rica	Four major hospitals in Costa Rica (Hospital San Juan de Dios, Hospital Calderón Guardia, Hospital Max Peralta and Hospital México)	Cohort study	To determine the influences of prognostic variables (age, sex, clinical stage, adjuvant therapy, type of dissection (D1 versus D2), extent of gastrectomy (partial versus total), margin status (R0 versus R1/2), tumor differentiation, and tumor location) on overall survival rates in a cohort of Hispanic patients after curative-intent surgery for gastric cancer	Patients who underwent surgery for advanced gastric cancer at four major hospitals in Costa Rica	236
Corral *et al* [6]	2015	Costa Rica; El Salvador; Guatemala; Honduras; Nicaragua; Panama	Costa Rica, El Salvador, Guatemala, Honduras, Nicaragua, Panama	Other: Summary analysis of cancer registries, hospital registries, and prospective population-based studies that used endoscopy and pathology registries.	To delineate the epidemiology of GC in Central America and contrast it with Hispanic-Latino populations in the U.S.	Central American patients who were native to Spanish-speaking Central American countries who were diagnosed with gastric cancer who were documented by the registries used to create this summary analysis	19,741
Dávila Meneses et al [19]	2018	Costa Rica	Max Peralta Hospital in Cartago Province	Case control study	To describe the clinical and epidemiological characteristics of patients screened at the Center for Early Detection of Gastric Cancer (CDTCG) at the Max Peralta Hospital in Cartago	Patients screened for gastric cancer at the Center for Early Detection of Gastric Cancer in Heredia, Costa Rica	903
Dominguez *et al* [20]	2019	Honduras	Western Honduras	Case control study	To examine the spatial epidemiology of gastric cancer subtypes and *H. pylori* virulence factors	Patients with gastric cancer documented in a registry within the Ministry of Health district hospital (Hospital de Occidente) of western Honduras in Santa Rosa de Copán, that serves as the principal referral center for the region.	702
Dominguez *et al* [21]	2013	Honduras	Western Honduras	Cross sectional study	To estimate the incidence of gastric cancer for Honduras using an endoscopy registry	Patients with non-cardia GC in western Honduras	670
Estevez-Ordonez *et al* [22]	2018	Honduras	Western Honduras	Case control study	To describe treatment patterns of gastric care in rural Honduras	Patients with gastric cancer who were treated in the time period between 2002 and 2015 in rural Western Honduras	741
Fantin *et al* [23]	2020	Costa Rica	Population-based, Costa Rica	Cross sectional study	To analyze the association between cancer incidence and socioeconomic position in Costa Rica between 2011 and 2015	Sample included all Costa Rican citizens aged 18 or older	3,138
Garita Acuña [24]	2017		Costa Rica	Cross sectional study	To identify the prevalence of the most common cancers diagnosed San Vicente de Paul Hospital	Patients who were diagnosed with cancer that were documented by San Vicente de Paul Hospital	
Leal-Mateos and Ortiz-Barboza [25]	2005	Costa Rica	Costa Rica	Cross sectional study	To analyze the mortality of cancer in elderly patients across Costa Rica in 2002	Patients in Costa Rica who died of any type of cancer in 2002	
Montalván *et al* [26]	2017	Honduras	Hospital de Occidente, Western Honduras	Cohort study	To estimate survival of gastric cancer in Honduras.	Patients diagnosed with gastric cancer at the Hospital de Occidente between 2002 and 2012	485
Mora Alvarado [27]	2003	Costa Rica	Costa Rica	Prevalence study	To describe changes in incidence rates and mortality of gastric cancer and their potential association with ecological factors in Costa Rica	Population-based study in Costa Rica	
Alvarado et al [28]	2006	Costa Rica	Costa Rica	Cross sectional study	To explore the relationship between gastric cancer incidence and mortality and nitrates in potable water in Costa Rica	458 districts in Costa Rica	
Norwood *et al* [29]	2021	Honduras	Western Honduras	Cancer registry	To report the first estimates of a PBCRs in Western Honduras	Western Honduras	
Rosero-Bixby and Sierra [30]	2007	Costa Rica	Cartago and Los Santos regions of Costa Rica	Other: Non-randomized community-control trial	To assess the validity and effect of gastric cancer X-ray screening on incidence, case survivorship, and mortality	Participants born between 1921 and 1945 were invited from the general population of Cartago and Los Santos.	87
Sasagawa *et al* [31]	1999	Costa Rica	Max Peralta Hospital in Cartago City, Costa Rica	Other: Description of a mass screening program	To report the progress of an X-ray mass screening program implemented by the Costa Rican and Japanese governments	Community members from the catchment area of Max Peralta Hospital in Cartago City, Costa Rica. 100% of men and 50% of women in this area were invited to participate.	43
Sasagawa *et al* [32]	2008	Costa Rica	Max Peralta Hospital in Cartago, Costa Rica	Cross sectional study	To present an analysis of extended lymph node (D2) dissection for gastric cancer patients compared to those in Japan	Gastric cancer patients treated at the Gastric Cancer Detection Center, Max Peralta Hospital, Cartago.	

**Table 3.** Calculated crude rates.

Author, Year	Country	Location	Start date	End date	Crude rate males	Crude rate females	Crude rate total
Costa Rica							
Barrantes Solís (2009) [15]	Costa Rica	San Vicente de Paúl Hospital in Heredia Province, Costa Rica	January 2000	August 2007			8.62/100,000
Cordero-García *et al* (2019) [17]	Costa Rica	Four major research hospitals in Costa Rica (Hospital San Juan de Dios, Hospital Rafael Ángel Calderón Guardia, Hospital Max Peralta and Hospital México)	January 2009	January 2012	1.04/100,000	0.77/100,000	0.90/100,000
Arevalo de Cordero-García *et al* (2018) [18]	Costa Rica	Four major research hospitals in Costa Rica (Hospital San Juan de Dios, Hospital Rafael Ángel Calderón Guardia, Hospital Max Peralta and Hospital México)	January 2009	January 2012	1.46/100,000	1.08/100,000	1.27/100,000
Dávila Meneses *et al* (2018) [19]	Costa Rica	Max Peralta Hospital in Cartago	1996	2015	10.33/100,000	8.79/100,000	9.56/100,000
Fantin *et al* (2020) [23]	Costa Rica	Country-wide	2011	2015			13.1/100,000
Alvarado *et al* (2006)* [28]	Costa Rica	Country-wide	1990	2000	35.63/100,000 (1990) 45.20/100,000 (1991) 36.83/100,000 (1992) 36.76/100,000 (1993) 37.32/100,000 (1994) 42.90/100,000 (1995) 41.41/100,000 (1996) 38.51/100,000 (1997) 37.59/100,000 (1998) 36.56/100,000 (1999) 35.22/100,000 (2000)	16.24/100,000 (1990) 19.22/100,000 (1991) 14.45/100,000 (1992) 16.06/100,000 (1993) 15.63/100,000 (1994) 21.18/100,000 (1995) 21.08/100,000 (1996) 19.77/100,000 (1997) 19.79/100,000 (1998) 18.84/100,000 (1999) 18.64/100,000 (2000)	25.94/100,000 (1990) 32.21/100,000 (1991) 25.64/100,000 (1992) 26.41/100,000 (1993) 25.98/100,000 (1994) 32.04/100,000 (1995) 31.25/100,000 (1996) 29.14/100,000 (1997) 28.69/100,000 (1998) 27.7/100,000 (1999) 26.93/100,000 (2000)
Rosero-Bixby and Sierra (2007) [30]	Costa Rica	Cartago and Los Santos	March 1996	June 2000	16.87/100,000	9.55/100,000	13.18/100,000
Sasagawa *et al* (1999) [31]	Costa Rica	Max Peralta Hospital in Cartago City, Costa Rica	March 1996	January 1998	25.0/100,000	7.62/100,000	16.28/100,000
Honduras							
Dominguez *et al* (2019) [20]	Honduras	Western Honduras	2002	2013			14.63/100,000
Dominguez *et al* (2013) [21]	Honduras	Western Honduras	2000	2009	28.1/100,000	14.0/100,000	16.75/100,000
Estevez-Ordonez *et al* (2018) [22]	Honduras	Western Honduras	2002	2015			13.23/100,000
Montalván *et al* (2017) [26]	Honduras	Hospital de Occidente, Western Honduras	2002	2012	15.74/100,000	8.10/100,000	11.88/100,000
Norwood *et al* (2021) [29]	Honduras	Western Honduras	January 2013	December 2017	18.62/100,000	10.84/100,000	20.10/100,000
El Salvador							
Arevalo de Acevedo and Arenivar Franco [14]	El Salvador	Country-wide	January 2000	December 2017			2.09/100,000
Panama							
Castro *et al* 2017 [16]	Panama	Country-wide	January 2012	December 2015	4.92/100,000	3.00/100,000	3.96/100,000
Multiple countries							
Corral *et al* 2015 [6]	Costa Rica; El Salvador; Guatemala; Honduras; Nicaragua; Panamá	Country-wide	1985	2011	All countries: 2.65/100,000 Guatemala: 0.62/100,000 El Salvador: 1.47/100,000 Honduras: 0.50/100,000 Nicaragua: 0.20/100,000 Costa Rica: 18.23/100,000 Panama: 1.06/100,000	All countries: 1.56/100,000 Guatemala: 0.52 /100,000 El Salvador: 1.00/100,000 Honduras: 0.27/100,000 Nicaragua: 0.15/100,000 Costa Rica: 10.22/100,000 Panama: 0.70/100,000	All countries: 2.10/100,000 Guatemala: 0.57/100,000 El Salvador: 1.22/100,000 Honduras: 0.39/100,000 Nicaragua: 0.18/100,000 Costa Rica: 14.25/100,000 Panama: 0.88/100,000

* authors provided crude rate directly in paper

**Table 4.** Stated risk factors and characteristics of gastric cancer.

Lead author	Year	Country	Number of gastric cancer cases	Listed risk factors	Anatomical site of cancer (Percent of patients)			Borrmann class (Percent of patients)						Histology (Percent of patients with each pathological subtype)		
					Cardia	Non-cardia	Unspecified	Type 1	Type 2	Type 3	Type 4	Type 5	Unknown	Intestinal	Diffuse	Indeterminant
Castro *et al* [16]	2017	Panama	611	This study found that lack of social security, a poorly differentiated tumor, clinical stage IV, and overlapping anatomic location with an increased risk of dying from GC. Insurance coverage, geographic location, and cultural differences were also listed as risk factors for mortality. Risk factors for having GC included male sex and older age.	11.3	39.1	3.6							53.7	35.6	10.6
Cordero-García *et al* [17]	2019	Costa Rica	168					3.6	10.7	48.8	16.1		20.8	87.5	12.5	
Cordero-García *et al* [18]	2018	Costa Rica	236			96.6	3.4							59.7	40.2	
Corral *et al* [6]	2015	Costa Rica; El Salvador; Guatemala; Honduras; Nicaragua; Panama	19,741	Male sex, living in rural areas	7	35	15.9							21.3	11.8	66.9
Dávila Meneses *et al* [19]	2018	Costa Rica	903	Age over 65, vomiting, loss of appetite, stomach pain, history of smoking, and antecedents of another type of cancer												
Dominguez *et al* [20]	2019	Honduras	702											52	48	
Dominguez *et al* [21]	2013	Honduras	670			100		0.9	9.6	60.3	7.2	15.1	7	33.8	56.3	9.9
Estevez-Ordonez et al [22]	2018	Honduras	741											45.2	43.2	
Fantin *et al* [23]	2020	Costa Rica	3,138	Lower socioeconomic status, living in a rural area												
Montalván *et al* [26]	2017	Honduras	485		28.25	62.06	9.96	1.03	4.95	66.19	9.28	14.43		43.6	43.39	10.74
Alvarado *et al* [28]	2003	Costa Rica		Genetics, age, diet, migration, altitude, environmental carcinogens, H. Pylori, Pteridium aquilinum												
Sasagawa *et al* [32]	2008	Costa Rica			21.1	70.8										

**Table 5.** Treatments and stated barriers to care.

Lead author	Year	Country	Treatments provided	Treatment Type (Percentage of Patients)				Stated barriers to care
				Surgery	Chemotherapy	Radiation	Palliative/ Supportive care	
Castro *et al* [16]	2017	Panama	Surgery; Chemotherapy; Radiation; Palliative/Supportive care	30.3	66.4	18	73.5	Lack of social security, geographic disparities (living in a remote region), cost of care
Cordero-García *et al* [17]	2019	Costa Rica	Surgery; Chemotherapy; Palliative/Supportive care	23.8	52.4		47.6	
Cordero-García *et al* [18]	2018	Costa Rica	Surgery; Chemotherapy; Radiation	100	17.8	39		
Dominguez *et al* [20]	2013	Honduras	Surgery					Living in an impoverished rural area, transportation difficulties, cultural issues, natural disasters, political turmoil
Estevez-Ordonez *et al* [22]	2018	Honduras	Surgery; Chemotherapy; Radiation; Palliative/Supportive care	22.9	8.5	2.6	17.3	Living in households below the regional poverty standard
Montalván *et al* [26]	2017	Honduras	Surgery; Chemotherapy; Radiation; Palliative/Supportive care	17.8	9.2		96.05	
Sasagawa *et al* [31]	1999	Costa Rica	Surgery	95.3				
Sasagawa *et al* [32]	2008	Costa Rica	Surgery	68.9				

**Table 6.** Mortality rates.

Lead author	Year	Country	Number of gastric cancer cases	Mortality rates		
				Males	Females	Total
Arevalo de Acevedo and Arenivar Franco [14]	2019	El Salvador	2,295	5 ± 1.6 / 100,000	2.5 ± 1.0 / 100,000	3.7 ± 1.2 / 100,000
Garita Acuña [24]	2017	Costa Rica				16% (mortality rate)
Leal-Mateos and Ortiz-Barboza [25]	2005	Costa Rica		6.9/100,000 (for males less than 65 years old)	113/100,000 (for females older than 65 years old)	
Mora Alvarado [27]	2003	Costa Rica		22.5/100,000 (1980) 22.2/100,000 (1981) 24.1/100,000 (1982) 27.0/100,000 (1983) 25.4/100,000 (1984) 26.6/100,000 (1985) 22.6 /100,000 (1986) 24.3/100,000 (1987) 27.4/100,000 (1988) 27.9/100,000 (1989) 25.5/100,000 (1990) 24.8/100,000 (1991) 25.8/100,000 (1992) 23.7/100,000 (1993) 23.3/100,000 (1994) 24.2/100,000 (1995) 26.2/100,000 (1996) 21.2/100,000 (1997) 20.1/100,000 (1998) 18.9/100,000 (1999)	10.1/100,000 (1980) 12.0/100,000 (1981) 10.7/100,000 (1982) 14.4/100,000 (1983) 13.8/100,000 (1984) 15.4/100,000 (1985) 15.2 /100,000 (1986) 13.3/100,000 (1987) 14.2/100,000 (1988) 15.0/100,000 (1989) 10.9/100,000 (1990) 13.8/100,000 (1991) 12.3/100,000 (1992) 13.7/100,000 (1993) 12.7/100,000 (1994) 14.0/100,000 (1995) 13.7/100,000 (1996) 10.3/100,000 (1997) 12.1/100,000 (1998) 11.1/100,000 (1999)	16.3/100,000 (1980) 17.1/100,000 (1981) 17.4/100,000 (1982) 20.7/100,000 (1983) 19.6/100,000 (1984) 21.0/100,000 (1985) 18.9/100,000 (1986) 18.8/100,000 (1987) 20.8/100,000 (1988) 21.4/100,000 (1989) 18.2/100,000 (1990) 19.3/100,000 (1991) 19.1/100,000 (1992) 18.7/100,000 (1993) 18.0/100,000 (1994) 19.1/100,000 (1995) 17.9/100,000 (1996) 15.7/100,000 (1997) 16.1/100,000 (1998) 15.0/100,000 (1999)
Alvarado *et al* [28]	2006	Costa Rica				71.0/100,000 (1971) 64.3/100,000 (1975) 62.6/100,000 (1980) 62.6/100,000 (1985) 62.3/100,000 (1990) 55.7/100,000 (1995) 45.7/100,000 (2000)
Rosero-Bixby and Sierra [30]	2007	Costa Rica	87			80/100,000

## Appendix 1. Final search strategies.

**PubMed**

((‘Central America’[MeSH Terms]) OR (‘central america*’[All Fields] OR ‘belize*’[All Fields] OR ‘costa rica*’[All Fields] OR ‘el salvador*’[All Fields] OR ‘salvadoran*’[All Fields] OR ‘salvadorian*’[All Fields] OR ‘salvadorean*’[All Fields] OR ‘guatemala*’[All Fields] OR ‘hondura*’[All Fields] OR ‘nicaragua*’[All Fields] OR ‘panama*’[All Fields])) AND (((stomach[All Fields] OR gastric[All Fields]) AND (neoplasms[All Fields] OR

cancer*[All Fields] OR carcinoma*[All Fields] OR neoplas*[All Fields] OR adenocarcinoma*[All Fields] OR tumour*[All Fields] OR tumour*[All Fields])) OR (‘stomach neoplasms’[MeSH])).

**Embase**

(‘stomach tumour’/exp OR ((stomach:au,ti,kw OR gastric:au,ti,kw) AND (cancer*:au,ti,kw OR carcinoma*:au,ti,kw OR neoplas*:au,ti,kw OR adenocarcinoma*:au,ti,kw))) AND (‘central america’/de OR ‘belize’/exp OR ‘costa rica’/exp OR ‘el salvador’/exp OR ‘guatemala’/exp OR ‘honduras’/exp OR ‘nicaragua’/exp OR ‘panama’/exp OR ‘central american’/de OR ‘belizean’/exp OR ‘costa rican’/exp OR ‘guatemalan’/exp OR ‘honduran’/exp OR ‘nicaraguan’/exp OR ‘panamanian’/exp OR ‘salvadoran’/exp OR ‘central america*’:ti,ab,kw OR belize*:ti,ab,kw OR ‘costa rica*’:ti,ab,kw OR ‘el salvador*’:ti,ab,kw OR salvadoran*:ti,ab,kw OR salvadorian*:ti,ab,kw OR salvadorean*:ti,ab,kw OR guatemala*:ti,ab,kw OR hondura*:ti,ab,kw OR nicaragua*:ti,ab,kw OR panama*:ti,ab,kw) NOT (‘conference abstract’/it OR ‘conference paper’/it OR ‘conference review’/it).

**Web of Science**

TS=((stomach OR gastric) AND (neoplas* OR cancer* OR carcinoma* OR adenocarcinoma*)) AND TS=(‘central america*’ OR belize* OR ‘costa rica*’ OR ‘el salvador*’ OR salvadoran* OR salvadorian* OR salvadorean* OR guatemala* OR hondura* OR nicaragua* OR panama*).

**Global Health (CAB Direct)**

(de:(‘Central America’ OR ‘Belize’ OR ‘Costa Rica’ OR ‘El Salvador’ OR ‘Guatemala’ OR ‘Honduras’ OR ‘Nicaragua’ OR ‘Panama’) OR (‘central america*’ OR belize* OR ‘costa rica*’ OR ‘el salvador*’ OR salvadoran* OR salvadorian* OR salvadorean* OR guatemala* OR hondura* OR nicaragua* OR panama*)) AND (de:(‘stomach cancer’) OR ((stomach OR gastric) AND (neoplas* OR cancer* OR carcinoma* OR adenocarcinoma*))).

**Latin America and Caribbean Health Sciences Literature**

(((stomach OR gastric) AND (neoplas$ OR cancer$ OR carcinoma$ OR adenocarcinoma$))) AND (((central america$) OR (belize$) OR (costa rica$) OR (el salvador$)) OR (salvadoran$ OR salvadorian$ OR salvadorean$ OR guatemala$ OR hondura$ OR nicaragua$ OR panama$)).
